# YTHDF3 modulates the progression of breast cancer cells by regulating FGF2 through m^6^A methylation

**DOI:** 10.3389/fcell.2024.1438515

**Published:** 2024-09-20

**Authors:** R. F. Gong, Z. H. Zhang, T. T. Sun, Y. X. Zhao, Wen Fang

**Affiliations:** ^1^ Center for Clinical Laboratories, The Affiliated Hospital of Guizhou Medical University, Guiyang, China; ^2^ School of Clinical Laboratory Science, Guizhou Medical University, Guiyang, China; ^3^ The Affiliated Cancer Hospital of Guizhou Medical University, The Affiliated Hospital of Guizhou Medical University, Guiyang, China

**Keywords:** breast caner, N6-methyladenosine, YTHDF3, FGF2, epigenetics

## Abstract

**Introduction:**

Breast cancer (BC) is a prevailing malignancy among women, and its inconspicuous development contributes significantly to mortality. The RNA N6-methyladenosine (m^6^A) modification represents an emerging mechanism for gene expression regulation, with the active involvement of the YTH N6-methyladenosine RNA binding protein 3 (YTHDF3) in tumor progression across multiple cancer types. Nonetheless, its precise function in breast cancer necessitates further investigation.

**Methods:**

The expression of YTHDF3 in both cell lines and patient tissues was examined using Western blotting, reverse transcription quantitative PCR (RT-qPCR), and immunohistochemistry (IHC) techniques. Bioinformatics analysis of methylated RNA immunoprecipitation sequencing (MeRIP-seq) and transcriptome RNA sequencing (RNA-seq) data was employed to screen for the target genes of YTHDF3. The main focus of this study was to investigate the *in vitro* biological functions of YTHDF3. The specific binding of YTHDF3 to its target genes and its correlation with m^6^A methylation were studied through RNA pull-down, RNA immunoprecipitation, and co-immunoprecipitation experiments. The protein regulatory mechanisms of downstream genes of YTHDF3 were assessed using protein stability analysis. Furthermore, the biological functions of YTHDF3 and its target genes in breast cancer cells were validated through CRISPR-Cas9 technology and rescue experiments.

**Results:**

By constructing a risk model using the TCGA database, YTHDF3 was identified as a high-risk factor among m^6^A methylation factors. Subsequent investigations revealed its elevated expression in various subtypes of breast cancer, accompanied by poor prognosis. MeRIP-seq analysis further revealed fibroblast growth factor 2 (FGF2) as a downstream gene of YTHDF3. Knockdown of YTHDF3 in breast cancer cells led to significant inhibition of cell self-renewal, migration, and invasion abilities *in vitro*. Mechanistically, YTHDF3 specifically recognized the methylated transcript of FGF2 within its coding sequence (CDS) region, leading to the inhibition of FGF2 protein degradation. Moreover, depletion of FGF2 markedly suppressed the biological functions of breast cancer cells, while reducing FGF2 expression in YTHDF3-overexpressing breast cancer cell lines substantially alleviated the malignant progression.

**Conclusions:**

In summary, our study elucidates the role of YTHDF3 as an oncogene in maintaining FGF2 expression in BC cells through an m^6^A-dependent mechanism. Additionally, we provide a potential biomarker panel for prognostic prediction in BC.

## 1 Introduction

N6-Methyladenosine (m^6^A) is the most prevalent and frequent internal post-transcriptional modification discovered in eukaryotic messenger RNAs (mRNAs). This crucial epigenetic component plays a significant role in regulating gene expression in a reversible manner. m^6^A is involved in the regulation of various cellular functions, including differentiation, self-renewal, invasion, and apoptosis (Bi et al., 2019; Lin et al., 2019; Liu L. et al., 2022). Moreover, exploring the impact of m^6^A modification on immune diseases reveals important mechanisms and therapeutic implications that could enhance our understanding of immune responses and potential treatments (Chen et al., 2024). The methylation of m^6^A is mediated by a methyltransferase complex that includes METTL3, METTL14, WTAP, VIRMA, RBM15, and ZC3H13, termed the “writers” of this modification. Conversely, m^6^A modification can be reversed by demethylases, specifically FTO and ALKBH5, termed “erasers” (Ma et al., 2019). The m^6^A modification is recognized by reader proteins that regulate RNA metabolism, including translation, splicing, export, and degradation (He et al., 2019). Therefore, m^6^A modifications are responsible for regulating the functions of mRNAs, microRNAs, and lncRNAs (Ma et al., 2019; Lin H. et al., 2022). Furthermore, the progression of m^6^A in the tumor microenvironment is influenced by factors such as hypoxia, immune responses, and metabolic reprogramming, which highlights its role in cancer biology (Han et al., 2024). Reader proteins containing the YT521-B homology (YTH) domain, such as YTHDF1-3, YTHDC1, and YTHDC2, specifically recognize m^6^A modifications and regulate m^6^A-modified mRNAs (Liao et al., 2018).

Numerous studies have demonstrated a strong association between m^6^A methylation and the progression of human cancers (Chen et al., 2019). For instance, FTO, an m^6^A demethylase, has been identified as a prognostic factor promoting cell proliferation and invasion in lung squamous cell carcinoma (LUSC) (Liu et al., 2018). Moreover, ALKBH5, another m^6^A demethylase, exhibits high expression in Glioblastoma stem-like cells (GSCs), and decreased expression of ALKBH5 in combination with FOXM1-AS impairs GSC tumorigenesis via the FOXM1 axis (Zhang et al., 2017). *In vitro* and *in vivo* studies have demonstrated the essential role of METTL3 in epithelial-mesenchymal transition (EMT) and metastasis in gastric cancer (Shi et al., 2017).

In 2020, breast cancer surpassed lung cancer and became the most prevalent malignancy worldwide (Sung et al., 2021). The identification of effective targets and the study of molecular mechanisms underlying breast cancer development and progression have become crucial areas of investigation in ongoing research. Breast cancer is a complex, multifactorial disease characterized by somatic gene mutations, copy number aberrations, exon sequencing alterations, changes in miRNA and protein expression levels, and DNA methylation modifications (Rahman et al., 2019). Epigenetic research has garnered increasing attention, offering new insights and avenues for studying breast cancer progression. By focusing on genetic and epigenetic differences, novel directions and innovative ideas for breast cancer research can emerge (Wang et al., 2023).

We initially observed that YTHDF3 expression was abnormally upregulated in breast cancer and played a crucial role in breast cancer cell growth and metastasis. Our analysis of multiple biomolecules revealed that fibroblast growth factor 2 (FGF2) is directly targeted by YTHDF3 in breast cancer cells. YTHDF3 regulates the translation of FGF2 in an m^6^A-dependent manner, influencing the malignant progression of breast cancer cells. Our data provide evidence that the m^6^A reader YTHDF3 plays a critical oncogenic role in the development of breast cancer.

## 2 Methods

### 2.1 Sources of the sequencing data

The RIP-seq and m^6^A-seq data for this study are available at NCBI GEO DataSets under accession numbers GSE130171 and GSE130172. The mRNA sequence data for shYTHDF3 are available at NCBI GEO DataSets under accession number GSE124817 (Chang et al., 2020).

### 2.2 Cell culture

293T and MCF-10A cells were obtained from the National Cell Resource Center (Shanghai, China). MCF-7 and MDA-MB-231 cells were acquired from Zhong Qiao Xin Zhou Biotechnology (Shanghai, China). All cell types were cultured in DMEM (GIBCO, United States) supplemented with 10% fetal bovine serum (FBS) (BI, United States) at 37°C with 5% CO_2_ in a cell incubator. All human cell lines have been authenticated using short tandem repeat (STR) or single nucleotide polymorphism (SNP) assays, and *Mycoplasma* scavengers (Plasmocin™ prophylactic, United States) are used regularly every 3 months to ensure that the cells are free of *mycoplasma* contamination.

### 2.3 Plasmids

To utilize shRNAs for lentivirus-mediated interference, complementary sense and antisense oligonucleotides encoding shRNAs targeting YTHDF3 were synthesized, annealed, and cloned into the pLKO.1 vector. To construct the YTHDF3 overexpression plasmid, the YTHDF3 gene was inserted into the pCDH-CMV-MCS-EF1-copGFP vector. To construct the YTHDF3-wild type (YTHDF1-FLAG) and YTHDF3-mutant (W438A, W492A) expression plasmids, these were cloned into the pCDH vector. To construct a knockdown FGF2 plasmid, it was synthesized to encode FGF2-targeting single guide RNAs (sgRNAs) that were annealed and subsequently cloned into lentiCRISPR v2 (#52961, Addgene). The synthesized shRNAs and sgRNA-related sequences are presented in Supplementary Table S1. pLKO.1 and pCDH-CMV-MCS-EF1-copGFP were obtained from IGE Biotechnology Ltd (Guangzhou, China).

### 2.4 Cell transfection and lentiviral infection

For transient transfection, Lipofectamine 3000 (Invitrogen, United States) was used to transfect cells with plasmid vectors. HEK293T cells were co-transfected with lentiviral vectors, including the packaging vectors psPAX2 (#12260, Addgene) and pMD2. G (#12259, Addgene), using Lipofectamine LTX (Invitrogen, United States) for lentivirus production. Infectious lentiviral particles were harvested from the cells 48 h post-transfection, filtered through a 0.45 μm PVDF filter, and then used to transfect other cells.

### 2.5 RNA isolation and RT-qPCR

Total RNA was extracted from cells and tissues using Trizol (Invitrogen, United States) according to the manufacturer’s instructions. In reverse transcription quantitative polymerase chain reaction (RT-qPCR), complementary DNA (cDNA) was synthesized from RNA using a Reverse Transcription Kit (Takara, Japan). The levels of RNA transcripts were analyzed using the Bio-Rad CFX96 real-time PCR system (Bio-Rad, United States). GAPDH was used to normalize all samples. Supplementary Table S1 contains all of the primers used in RT-qPCR.

### 2.6 Western blot and Co-IP

MCF-7 or MDA-MB-231 cells were rinsed twice with cold phosphate-buffered saline (PBS) and then centrifuged. Afterward, the pellet was resuspended in lysis buffer and incubated on ice with frequent vortexing for 10 min. Finally, the lysate was obtained by centrifugation at 12,000 g for 10 min. SDS-polyacrylamide gel electrophoresis (SDS-PAGE) was used to fractionate the proteins, which were then transferred onto polyvinylidene fluoride (PVDF) membranes. The membranes were then blocked with 5% non-fat milk in TBST and subsequently blotted with specific antibodies. The antibodies used included anti-YTHDF3 (1:2000, Abcam), anti-GAPDH (1:10000, Abcam), anti-FGF2 (1:2000, Abcam), and anti-FLAG-tag (1:1000, Sigma-Aldrich).

For co-immunoprecipitation (co-IP), cell lysates containing 1 × 10^7^ cells were immunoprecipitated with IP buffer containing agarose beads coupled to specific antibodies. Protein-protein complexes were detected by Western blotting. IgG was used as a negative control.

### 2.7 Cell growth and proliferation assay

Cell viability was assessed by adding 10% CCK-8 (DOJINDO, Japan) to infected cells in 96-well plates. The cells were then incubated at 37°C for 2 h at 0, 24, 48, 72, and 96 h. All experiments were performed in triplicate.

For colony formation assays, each well of a six-well plate was seeded with 1 × 10^3^ infected MCF-7 or MDA-MB-231 cells, and the medium was replaced every 3 days. After 10 days, the colonies were fixed with paraformaldehyde, stained with 0.1% crystal violet (Solarbio, China) for 30 min, and then rinsed with PBS. Colonies containing more than 50 cells were counted.

### 2.8 Cell migration and invasion assay

Migration assays were performed using a 24-well Transwell chamber system (Corning, United States). Breast cancer cells were seeded in the upper chamber of a 24-well Transwell insert, which contained 0.4 mL of serum-free medium. The lower chamber was filled with 0.6 mL of medium containing 20% FBS. After a 24-h incubation, the cells were fixed with paraformaldehyde for 15 min and subsequently stained with 0.1% crystal violet (Solarbio, China) for 30 min. After rinsing with water, the chamber membranes were mounted onto slides.

For the invasion assay, Matrigel (BD, United States) was applied to the upper chamber of the 24-well Transwell inserts before seeding the cells. Migrated or invaded cells were imaged and counted under a 20 × microscope.

### 2.9 Apoptosis assay

Cellular staining was performed according to the manufacturer’s guidelines using the Cell Apoptosis Assay Kit (Multisciences, China). Subsequently, flow cytometric analysis of the stained cells was conducted using a BD flow cytometer.

### 2.10 RNA immunoprecipitation (RIP)

The cells were washed twice in PBS, harvested, and resuspended in IP lysis buffer (150 mM KCl, 25 mM Tris (pH 7.4), 5 mM EDTA, 0.5 mM DTT, 0.5% NP-40, 1× protease inhibitor, and 1 U RNase inhibitor). After a 30-min incubation, the lysate was centrifuged at 12,000 g for 10 min. Subsequently, the lysate was incubated with antibodies and 40 μL of protein G beads (Thermo Fisher, United States) overnight at 4°C. After three washes with the washing buffer (150 mM KCl, 25 mM Tris (pH 7.4), 5 mM EDTA, 0.5 mM DTT, 0.5% NP-40), RNA co-precipitated with the lysate was extracted using Trizol reagent, and ethanol was used for glycogen precipitation (Invitrogen, United States). The degree of RNA enrichment was normalized against that of IgG.

### 2.11 Vector and m^6^A mutation assays

The potential m^6^A sites were predicted using an online tool, SRAMP (http://www.cuilab.cn/sramp/). The FGF2 CDS region, and the m^6^A motif depleted CDS regions were cloned into pcDNA3.1 for the RNA pull-down assay.

### 2.12 RNA pull-down

The RNA pull-down assay was performed using the Pierce Magnetic RNA-Protein Pull-down Kit (Thermo Fisher Scientific). Specifically, biotin-labeled FGF2-WT, FGF2-Mut, YTHDF3, and YTHDF3-Mut RNA were used in the assay. MCF-7 cell lysates were incubated with biotin-labeled RNA bound to magnetic beads at 4°C overnight. After washing with the appropriate buffer, the complexes were purified. Relevant proteins were detected by Western blot analysis.

### 2.13 Protein stability

Protein stability of breast cancer cells was evaluated by treating them with 100 μg/mL CHX and 10 µM MG132 for the indicated time intervals. The cells were then harvested. Protein expression of either FGF2 or YTHDF3 were determined by Western blot analysis.

### 2.14 Statistics

Analyses were conducted using Linux, the R platform, and GraphPad Prism 8. Statistical analyses were carried out using the R platform. The relevant data were derived from three independent replicate experiments. Results are expressed as mean ± standard deviation. Differences between the two groups were analyzed using a paired two-tailed Student’s t-test, one-way ANOVA, and chi-square tests

## 3 Results

### 3.1 YTHDF3 is highly expressed in BRCA and associated with poor prognosis

**FIGURE 1:**
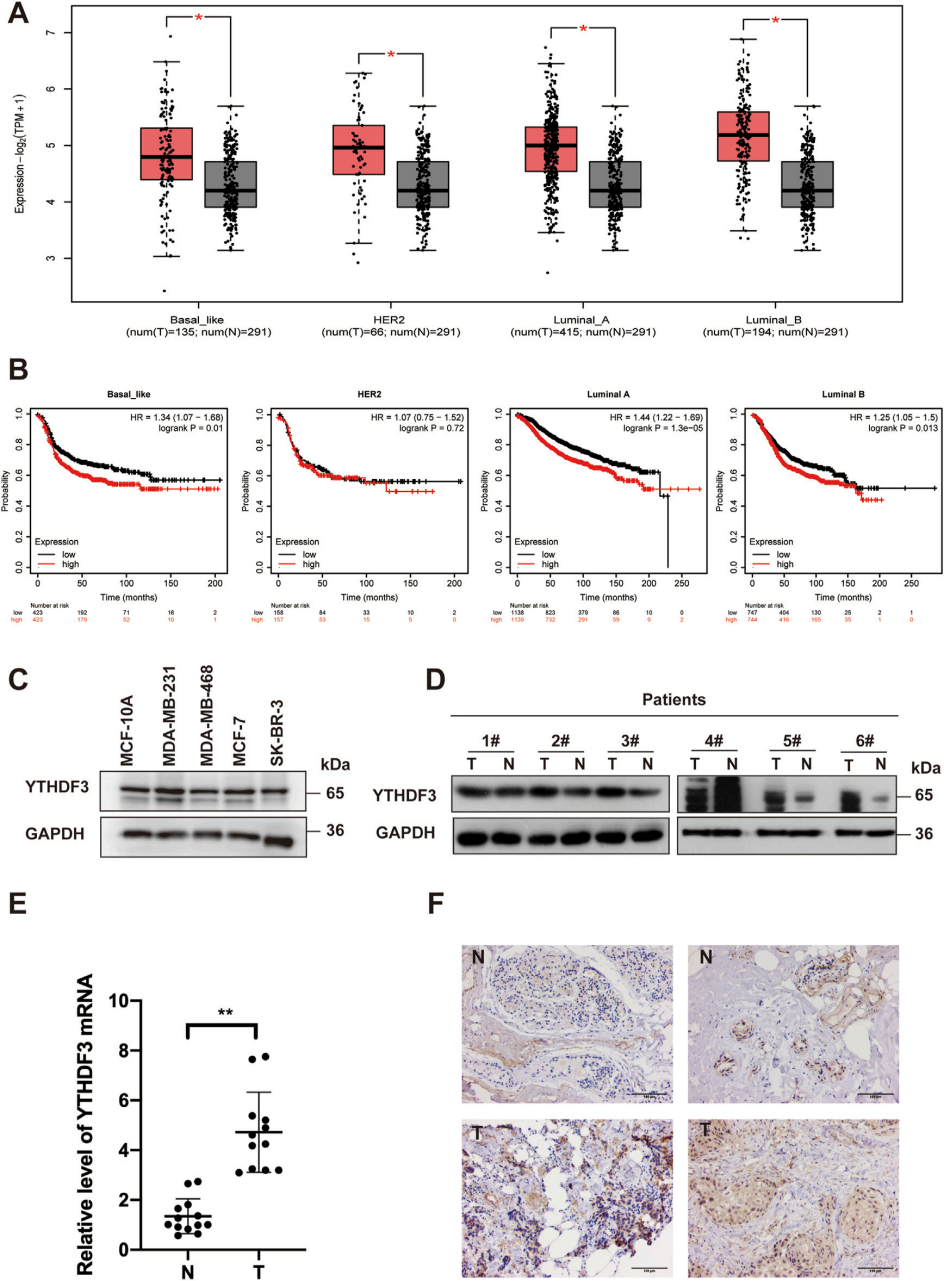
The overexpression of YTHDF3 is associated with an unfavorable prognosis in breast cancer patients. **(A)** Analysis of YTHDF3 expression across different PAM50 subtypes of breast cancer using the GEPIA dataset. **(B)** Survival analysis illustrating the impact of YTHDF3 expression on the survival rates of breast cancer patients with distinct PAM50 subtypes. **(C)** Protein levels of YTHDF3 in both breast cancer and normal breast surface epithelial cells. **(D)** Comparative assessment of YTHDF3 protein levels in breast cancer patient tissues and normal breast tissues. **(E)** Comparative analysis of YTHDF3 mRNA levels in breast cancer tissues and normal breast tissues, with data represented as mean ± standard deviation (S.D). **(F)** Representative immunohistochemical images depicting the representative immunostaining of YTHDF3 in breast cancer tissues and normal breast tissues. Scale bar is set at 100 μm (N:Normal tissue,T:Tumor tissue).

To assess the risk factors associated with m^6^A regulators in breast cancer (BRCA), we analyzed data from The Cancer Genome Atlas (TCGA) database. Our analysis highlighted the significance of YTH N6-methyladenosine RNA-binding protein (YTHDF3) in constructing a risk model for breast cancer (Supplementary Figure S1). Notably, YTHDF3 displayed significant expression and correlation with various subtypes of breast cancer in the TCGA database (Figure 1A). Although patients with high expression of YTHDF3 in the HER^2+^ subtype did not show a significant survival difference compared to those with low expression, overall, patients with elevated YTHDF3 expression consistently demonstrated poorer survival rates. Furthermore, elevated YTHDF3 expression levels were consistently associated with poorer survival outcomes (Figure 1B). These distinct expression patterns prompted further investigation into the functional and clinical implications of YTHDF3 in BRCA. In addition, all breast cancer cell lines, except the HER2+ cell line SK-BR-3, exhibited increased levels of YTHDF3 expression compared to the normal mammary epithelial cell line MCF-10A (Figure 1C). Moreover, both YTHDF3 protein and mRNA levels were significantly elevated in most BRCA patient tissues compared to normal tissues (Figures 1D, E). To evaluate the clinical significance of YTHDF3 in BRCA, we performed immunohistochemical (IHC) staining for YTHDF3 on tissue samples obtained from Guizhou Provincial Tumor Hospital (Figure 1F). Our findings demonstrated intensified YTHDF3 staining, indicative of increased malignancy. Collectively, these results suggest that the m^6^A reader YTHDF3 is overexpressed in breast cancer and is associated with poor prognosis in breast cancer patients

### 3.2 YTHDF3 plays an oncogenic role in breast cancer cells

**FIGURE 2:**
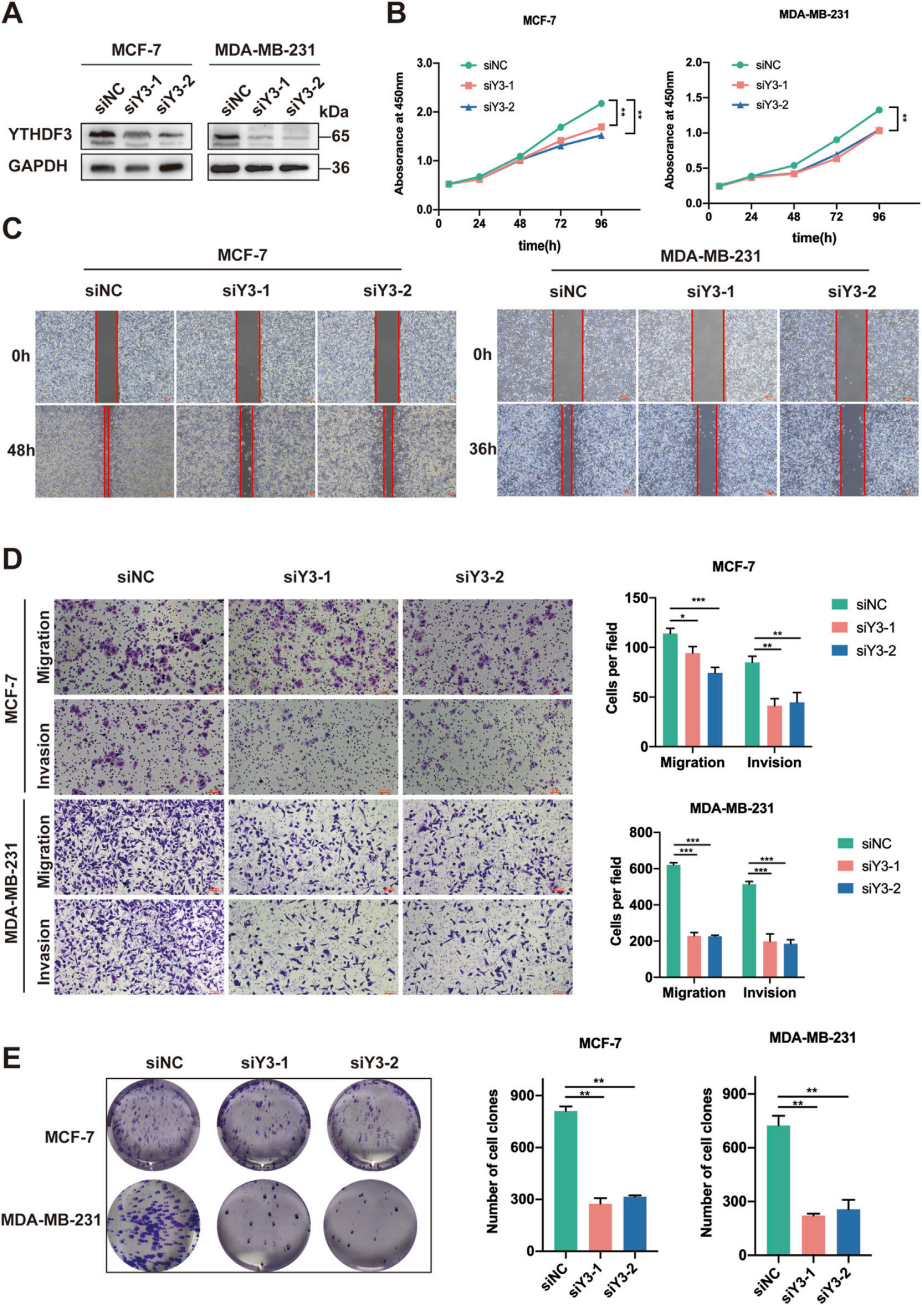
Suppression of YTHDF3 Inhibits *In Vitro* Growth and Migration of Breast Cancer Cells. **(A)** Western blot analysis was conducted to assess the expression of YTHDF3 in MCF-7 and MDA-MB-231 cells transfected with two independent siRNAs specifically targeting YTHDF3 or control siRNA. **(B)** Cell growth was determined through CCK8 assay following YTHDF3 knockdown in MCF-7 and MDA-MB-231 cells. **(C)** Scratch assay was performed on MCF-7 and MDA-MB-231 cells as described in **(A)**. **(D)** YTHDF3 knockdown led to reduced migration and invasion capabilities of MCF-7 and MDA-MB-231 breast cancer cells. **(E)** Colony formation assay was conducted on MCF-7 and MDA-MB-231 cells. *p < 0.05, **p < 0.01, ***p < 0.001.

To elucidate the role of YTHDF3 in breast cancer, our study aimed to investigate the cellular changes that occur in breast cancer cells following YTHDF3 knockdown. Successful knockdown of YTHDF3 was achieved in MCF-7 and MDA-MB-231 breast cancer cells using siRNAs (siY3-1 and siY3-2) (Figure 2A). Moreover, CCK-8 assays demonstrated a significant impairment in cell growth following YTHDF3 knockdown in both MCF-7 and MDA-MB-231 cells (Figure 2B). Furthermore, the wound healing assay revealed a notable decrease in the scratch healing ability of breast cancer cells after YTHDF3 knockdown (Figure 2C). Additionally, the cell colony formation assay exhibited a significant inhibition of the ability of cells to form colonies after YTHDF3 knockdown (Figure 2E). Moreover, the cell migration and invasion assays indicated that YTHDF3 knockdown impaired the migration and invasion capabilities of both MCF-7 and MDA-MB-231 cells. Collectively, these findings highlight the pivotal role of YTHDF3 in the proliferation, migration, and invasion of breast cancer cells, suggesting its potential as a pro-oncogenic factor in breast cancer progression.

### 3.3 Identification of FGF2 as a target of YTHDF3 in breast cancer

In order to uncover the underlying mechanisms of YTHDF3 in breast cancer occurrence and development, we first analyzed conducted an analysis of RNA-seq datasets of YTHDF3 knockout cells and control cells available in the literature (Chang et al., 2020). YTHDF3 knockout resulted in comprehensive alterations in gene expression (Supplementary Figure S2A). Gene Ontology (GO) analysis revealed enrichment in several pathways, including the MAPK signaling pathway, VEGF signaling pathway, and protein processing in the endoplasmic reticulum (Supplementary Figure S2B). Additionally, gene set enrichment analysis (GSEA) demonstrated that the genes affected by YTHDF3 were associated with MicroRNAs in cancer, Lipid and atherosclerosis, MAPK signaling pathway, and protein processing in the endoplasmic reticulum (Supplementary Figures S2C–F), further supporting the regulatory role of YTHDF3 in breast cancer tumorigenesis.

YTHDF3, a widely recognized m^6^A “readers”, exerts its function by interacting with and modulating m^6^A methylated transcripts. Analysis of meRIP-seq data from the literature15 revealed an m^6^A binding domain between FGF2 and YTHDF3, as depicted in the IGV plot (Supplementary Figures S2G, H). This finding suggests that FGF2 could potentially be a downstream target of YTHDF3, subject to regulation through m^6^A methylation. To further investigate the relationship between FGF2 and YTHDF3, RNA immunoprecipitation followed by quantitative PCR (RIP-qPCR) confirmed the interaction between YTHDF3 and FGF2 mRNA in MCF-7 and MDA-MB-231 breast cancer cells (Figure 4F).

### 3.4 YTHDF3 enhances FGF2 protein stability via an m^6^A-dependent manner

**FIGURE 3:**
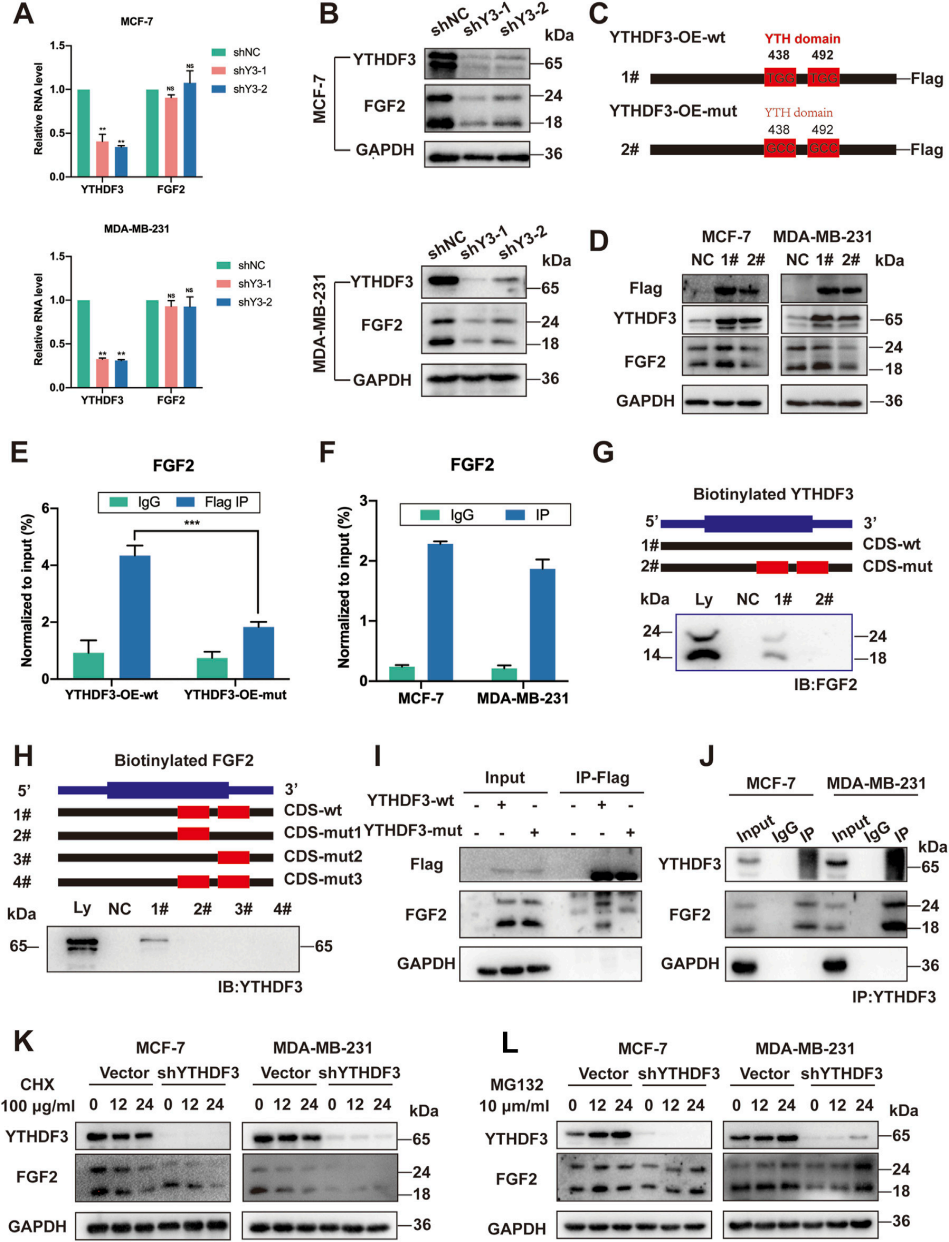
YTHDF3 Controls FGF2 Translation in an m^6^A-Dependent Manner. **(A)** Assessment of relative RNA levels of FGF2 in MCF-7 and MDA-MB-231 cells upon YTHDF3 knockdown. **(B)** Western blot analysis conducted to determine FGF2 protein levels in MCF-7 and MDA-MB-231 cells following YTHDF3 knockdown. **(C)** Schematic representation of YTHDF3 constructs, including wild-type (YTHDF3-OE-wt) and mutant-type (YTHDF3-OE-mut). **(D)** Western blot analysis performed to evaluate FGF2 and Flag-tagged protein levels in MCF-7 and MDA-MB-231 cells after YTHDF3 overexpression and mutation. **(E)** RT-qPCR analysis performed after YTHDF3 RIP validated the interaction between YTHDF3 and FGF2 mRNA. **(F)** RIP followed by RT-qPCR using Flag-tag targeting, after YTH domain mutation, confirmed the interaction between YTHDF3 and FGF2 mRNA mediated by the YTHDF3 domain. **(G)** To investigate the interaction of FGF2, immunoblot analysis was performed in MCF-7 cells using cellular lysates (Ly.), biotin-labeled YTHDF3 coding sequence (CDS) region with or without YTH domain (#1, #2), and the bead-only control (NC) through immunoprecipitation. **(H)** Immunoblot analysis was conducted in MCF-7 cells to assess the interaction of YTHDF3 with cellular lysates (Ly.), full-length biotinylated FGF2 (#1), FGF2 coding sequence (CDS) regions with predicted single m^6^A gene mutations (#2, #3), FGF2 region with predicted mutations in all m^6^A genes (#4), and the bead-only control (NC). **(I)** Co-IP was carried out in MCF-7 cells to examine the influence of the YTH domain in YTHDF3 on its interaction with FGF2, using the Flag tag. **(J)** Reverse Co-IP was performed to affirm the mutual interaction between FGF2 and YTHDF3 **(K, L)** Treatment of NC and shYTHDF3 cells with CHX and MG132 was performed for the specified time. Then, Western blotting was conducted on cell extracts to analyze the degradation rate of FGF2 protein, GAPDH was used as a loading control for protein. Data are shown as means ± S.D. *p < 0.05, **p < 0.01, ***p < 0.001, ns, not significant.

Previous studies have implicated the YTH family in specific roles in controlling the fate of mRNA methylation (Yang et al., 2023; Zhao et al., 2020). To elucidate the specificity of FGF2 as an m^6^A reader and determine its m^6^A-dependent regulatory mechanism, we first examined the transcription and translation of FGF2 following YTHDF3 depletion. As expected, silencing YTHDF3 with shRNAs in MCF-7 and MDA-MB-231 breast cancer cells reduced in FGF2 protein levels without affecting its RNA levels (Figures 3A, B). The decrease in FGF2 protein levels but not mRNA levels in the absence of YTHDF3 led us to speculate that YTHDF3 may regulate the stability or translation efficiency of FGF2 protein. To validate this, control or YTHDF3-depleted breast cancer cells were treated with the protein translation inhibitor cycloheximide (CHX). Western blot analysis revealed enhanced stability of FGF2 protein in YTHDF3 knockout MCF-7 and MDA-MB-231 cells. Therefore, we discovered that YTHDF3 functions by enhancing FGF2 protein stability. Next, we investigated whether the regulation of FGF2 expression by YTHDF3 depends on m^6^A methylation. It is known that YTHDF3 can bind to m^6^A sites through its m^6^A-binding domain in the YTH domain, and mutations at W438 and W492 weaken the binding ability of YTHDF3 with mRNA (Chang et al., 2020). We introduced point mutations W438A and W492A in the YTH domain of FLAG-tagged YTHDF3 (YTHDF3-OE-mut) and transfected breast cancer cells with constructs expressing either wild-type YTHDF3 (YTHDF3-OE-wt) or mutant YTHDF3 (YTHDF3-OE-mut) (Figure 3C). Subsequently, RNA immunoprecipitation (RIP) using FLAG antibody followed by qPCR demonstrated that cells transfected with YTHDF3-wt effectively immunoprecipitated FGF2 mRNA, while the interaction between YTHDF3 mutant and FGF2 mRNA was significantly reduced (Figure 3E), indicating the crucial importance of the m^6^A-binding domain for the interaction between YTHDF3 and FGF2 mRNA. Importantly, we observed that YTHDF3-OE-wt, but not YTHDF3-OE-mut, increased the protein expression of FGF2 (Figure 3D). Notably, RNA pull-down assays revealed that FGF2 primarily binds to the coding sequence (CDS) region of YTHDF3 in MCF-7 cells, and this binding was significantly weakened when the m^6^A-binding region was deleted (Figure 3G). Reverse pull-down experiments with mutations in the predicted m^6^A-binding domain of FGF2 showed a significant reduction in binding when the m^6^A-binding domain of FGF2 was mutated (Figure 3H), further validating the role of YTHDF3 in regulating FGF2 protein expression through m^6^A methylation. Co-immunoprecipitation (Co-IP) and Western blot analysis using Flag-tagged YTHDF3 demonstrated a significant reduction in binding between FGF2 and YTHDF3 when the m^6^A-binding domain was mutated (Figure 3I). Furthermore, reverse FGF2 pull-down experiments confirmed the interaction between YTHDF3 and FGF2 and its regulation through m^6^A methylation (Figure 3J).

In summary, our data demonstrate that the FGF2 transcript is directly recognized by the m^6^A “reader” YTHDF3. FGF2 maintains transcript stability through an m^6^A-YTHDF3-dependent mechanism, preventing degradation and naturally increasing its expression.

### 3.5 The downregulation of FGF2 inhibits the malignant progression of breast cancer cells

**FIGURE 4:**
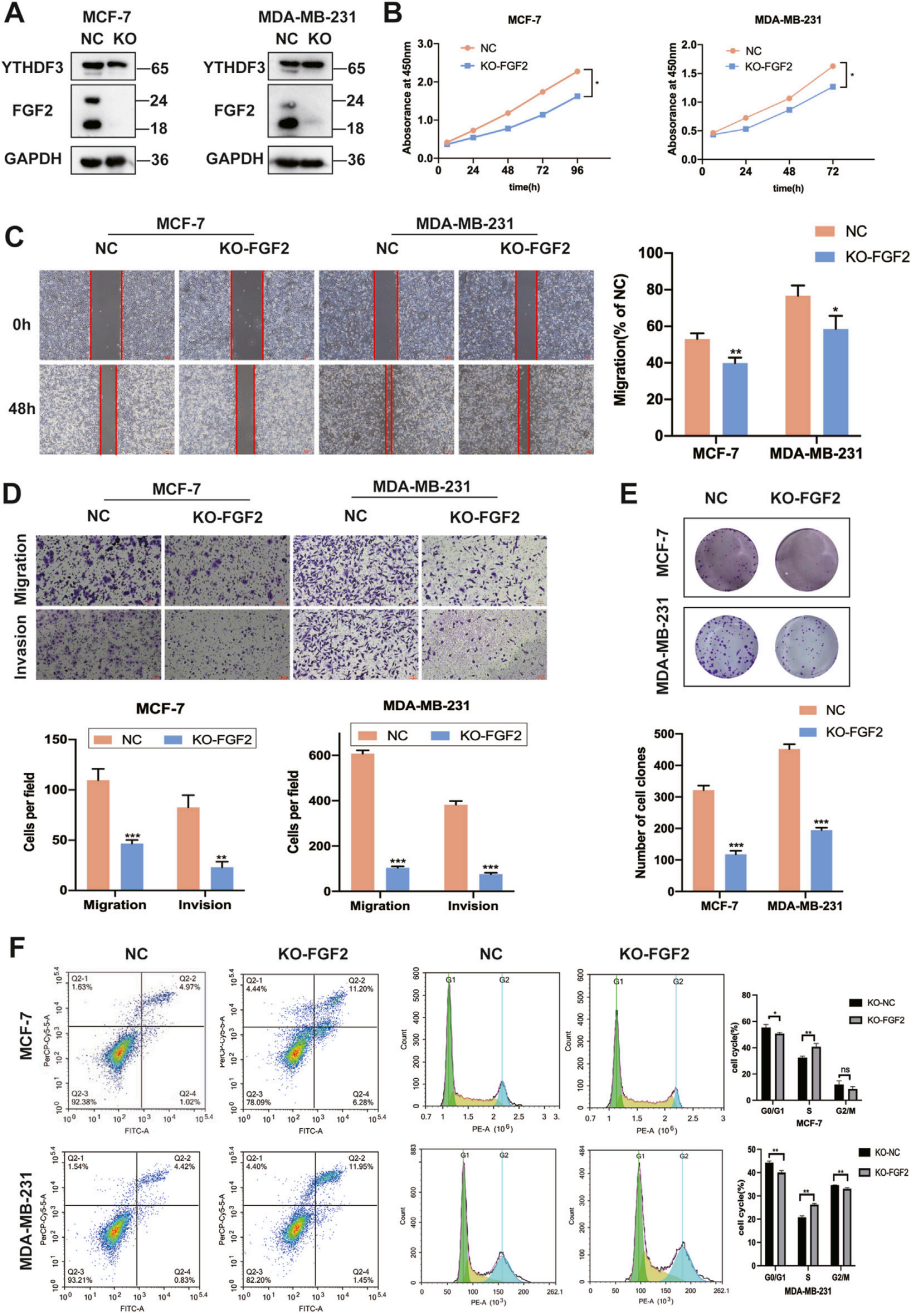
Downregulation of FGF2 can inhibit the malignant progression of breast cancer cells. **(A)** Western blotting was employed to assess the expression of YTHDF3 and FGF2 in MCF-7 and MDA-MB-231 cells following targeted CRISPR-Cas9 knockout of FGF2 **(B)** Cell growth was evaluated using the CCK-8 assay after FGF2 knockout in MCF-7 and MDA-MB-231 cells. **(C)** Scratch assays were conducted on MCF-7 and MDA-MB-231 cells according to the aforementioned protocol **(D)** Deletion of the FGF2 gene resulted in attenuated migration and invasion abilities in MCF-7 and MDA-MB-231 breast cancer cells. **(E)** Colony formation assays were performed on MCF-7 and MDA-MB-231 cells **(F)** Apoptosis and cell cycle analysis were carried out on the FGF2 knockout cell lines. *p < 0.05, **p < 0.01, ***p < 0.001, ns, not significant.

Due to the ambiguous role of FGF2 in breast cancer cells, we first employed CRISPR-Cas9 technology to target and knock out FGF2. Western blot analysis revealed that the expression of YTHDF3 was not significantly altered following FGF2 knockout (Figure 4A). Notably, the cell growth and colony formation abilities of these two breast cancer cell lines were significantly diminished following FGF2 knockout (Figures 4B, E). Furthermore, the loss of FGF2 re-induced the migration and invasion of breast cancer cells (Figure 4D). Concurrently, scratch assays indicated that the wound healing ability was diminished after FGF2 knockout (Figure 4C). To further investigate the impact of FGF2 on the cell cycle of breast cancer cells, flow cytometry analysis revealed that FGF2 knockout arrested MCF-7 and MDA-MB-231 cells in the S phase and induced early apoptosis in both cell lines (Figure 4F). These findings suggest that the downregulation of FGF2 inhibits tumorigenesis in breast cancer cells.

**FIGURE 5:**
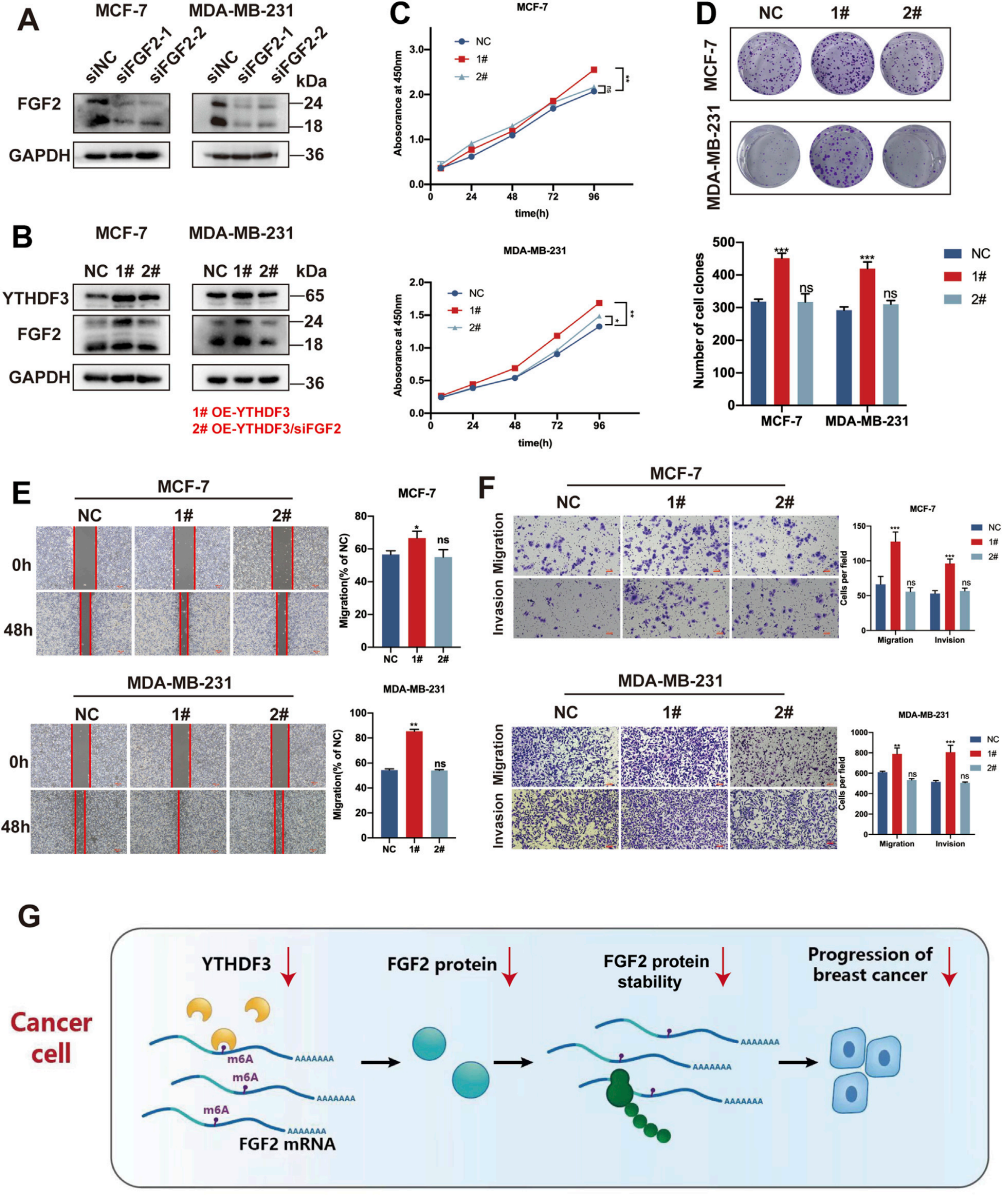
YTHDF3 facilitates the growth and migration of breast cancer cells through its dependence on FGF2. **(A)** Relative protein expression of two siRNAs targeting FGF2 was assessed through Western blot **(B)** Western blot analysis of YTHDF3 and FGF2 protein levels in MCF-7 and MDA-MB-231 cells with FGF2 silenced by YTHDF3 overexpression. **(C)** Cell growth was detected in MCF-7 and MDA-MB-231 cells from **(B)** using CCK8 assay. **(D)** Clone formation assays were conducted to validate the proliferative capacity of MCF-7 and MDA-MB-231 cells from **(B)**. **(E)** Scratch assays were performed to measure the wound healing ability of MCF-7 and MDA-MB-231 cells from **(B)**. Scale bar, 100 μm. **(F)** Transwell assays were used to measure the migration and invasion abilities of MCF-7 and MDA-MB-231 cells from **(B)**. Scale bar, 100 μm. **(G)** Proposed model illustrating the role of YTHDF3-mediated FGF2 translation in breast cancer. NC (negative control), 1# (OE-YTHDF3), 2# (OE-YTHDF3/siFGF2) *p < 0.05, **p < 0.01, ***p < 0.001. ns, not significant.

The protein blotting results indicated that siFGF2-1 exhibited the most potent silencing effect. Therefore, siFGF2-1 was selected for subsequent experiments (Figure 5A). We knocked down the expression of FGF2 in MCF-7 and MDA-MB-231 cells overexpressing YTHDF3, and observed alterations in FGF2 levels in both cell lines (Figure 5B). For subsequent cellular biology experiments, we established the following groups: NC (negative control), 1# (OE-YTHDF3), and 2# (OE-YTHDF3/siFGF2). Overexpression of YTHDF3 promoted cell growth and colony formation, whereas downregulation of FGF2 reversed these effects (Figures 5C, D). Similarly, following FGF2 knockdown,the enhanced cell growth and invasion caused by YTHDF3 overexpression were suppressed (Figures 5E, F). These findings indicate that FGF2 is an important downstream target through which YTHDF3 promotes breast cancer progression (Figure 5G).

## 4 Discussion

The most prevalent mRNA modification, N6-methyladenosine (m^6^A), surpasses others in terms of abundance. On average, every 1000 nucleotides harbors 1-2 m^6^A residues. This modification predominantly occurs in eukaryotic cells, specifically at the RRACH sequence (with R = A or G, H = A, C, or U) (Krug et al., 1976; Beemon and Keith, 1977; Bokar et al., 1997). Remarkably, m^6^A methylation has been detected not only in eukaryotic cells but also in viruses, prokaryotic cells, and even viral genomes, highlighting its universal presence (Dai et al., 2018). Mechanistically, m^6^A methylation is intricately involved in numerous facets of RNA metabolism, encompassing mRNA translation, degradation, splicing, export, and folding (Liu and Gregory, 2019; He and He, 2021). Despite its widespread existence throughout eukaryotes, the role of m^6^A methylation in cancer is nuanced and cannot be simply categorized as “beneficial” or “detrimental” (Melstrom and Chen, 2020).

YTHDF3, a member of the YTH family characterized by the presence of the YTH domain, exhibits highly conserved genetic features across various animal and plant species, underscoring its undeniable significance (Chen et al., 2023). The YTH family comprises five known members: YTHDF1, YTHDF2, YTHDF3, YTHDC1, and YTHDC2, all of which are involved in m^6^A modification and function as “readers” of this modification. As integral components of the “reading” machinery, they play a crucial role by recognizing and binding to m^6^A-modified sites, thereby modulating the function of target RNAs (Chen et al., 2019; Xu et al., 2021). For instance, YTHDF1 inhibits the presentation of tumor neoantigens to T cells by recognizing m^6^A modification in dendritic cells, leading to tumor cell evasion from immune surveillance (Han et al., 2019). YTHDF2 interacts with miRNAs and influences the migration and invasion capabilities of prostate cancer cells (Li et al., 2018). YTHDF3, in concert with YTHDF2 and YTHDF1, contributes to translational regulation. Furthermore, YTHDF3 recognizes the m^6^A-modified initiation factor eIF4G2 and participates in the translation process of circular RNAs (CircRNAs). In addition, YTHDF3 affects the occurrence and progression of colorectal cancer through the regulation of m^6^A modification (Ni et al., 2019). YTHDF3 also plays a role in immune-related microenvironments by promoting FOXO3 translation, thereby suppressing interferon-dependent antiviral responses. In conclusion, “Readers” holds immense potential in cancer research and serves as a promising avenue for exploring m^6^A modification, thereby warranting further investigation.

According to reports, YTHDF3 plays a pivotal role as a crucial component of m^6^A methylation across various tumor types (Anita et al., 2020; Du et al., 2023; Lin Y. et al., 2022; Xu et al., 2022). Previous studies have also emphasized the involvement of YTHDF3 in brain metastasis of breast cancer cells (Chang et al., 2020), as well as its significant role in the progression and metastasis of triple-negative breast cancer through the YTHDF3-ZEB1 axis (Lin Y. et al., 2022). In our research, we observed upregulation of the YTHDF3 gene in breast cancer, indicating an increased risk. Additionally, in several intrinsic molecular subtypes of breast cancer, heightened expression of YTHDF3 is accompanied by increased patient mortality rates, suggesting its importance as an oncogene in breast cancer and its selection during the cancer evolution process. Our findings align with a previous study (Anita et al., 2020; Liu J. et al., 2022). Furthermore, our research reveals the pronounced promotion of malignant processes, including proliferation, migration, and invasion, by YTHDF3 in breast cancer cells. These findings provide insightful revelations, highlighting towards the potential of YTHDF3 as a promising therapeutic target in the treatment of breast cancer.

To unravel the underlying mechanisms of YTHDF3 in breast cancer, we conducted a comprehensive analysis using bioinformatics tools and examined previously published meRIP-Seq and RIP-Seq data (Chang et al., 2020). Our primary objective was to identify potential downstream targets of YTHDF3 that could be modulated through m^6^A methylation. The bioinformatics analysis highlighted FGF2 as a promising candidate downstream target of YTHDF3, with plausible involvement in m^6^A methylation regulation. However, the regulatory relationship between YTHDF3 and FGF2 necessitates further deliberation. To investigate this, we performed RIP experiments in breast cancer cell lines, which revealed an interaction between FGF2 and YTHDF3 mRNA. However, the precise regulatory mechanism involving YTHDF3-induced m^6^A methylation modifications remains to be elucidated. Further research is warranted to clarify this aspect.

Previous studies have predominantly explored FGF2 as a target within the FGF2/FGFR1 signaling pathway, employing exogenous FGF2 to facilitate breast cancer progression (Santolla et al., 2019). Interestingly, it has been observed that the effects of exogenous FGF2 differ significantly from those of intracellular overexpression of FGF2 in breast cancer cells (Korah et al., 2000). Consequently, FGF2 presents a paradoxical situation in breast cancer cells. In our investigation, we employed CRISPR-Cas9 technology to specifically knock out FGF2. The depletion of FGF2 in breast cancer cell lines notably attenuated their migration, invasion, and clonogenic capacities, while also inducing apoptosis in these cells, echoing findings from a previous study (Maloof et al., 1999). These conflicting observations further fuel our curiosity about whether YTHDF3 influences the malignant processes of breast cancer cells by regulating FGF2 expression.

To gain further insights into the YTHDF3-m^6^A-FGF2 axis, we introduced specific mutations in the YTHDF3 protein, specifically altering the hydrophobic residues W438 and W492 to alanine. These mutations are known to disrupt the YTH domain, thus impairing the m^6^A binding capability. Subsequently, we generated plasmids containing the mutated sequences and conducted RIP-qPCR and RNA pull-down experiments to confirm the direct interaction between FGF2 mRNA and the YTH domain of YTHDF3. Interestingly, overexpression of the mutated plasmids did not affect FGF2 protein levels, and silencing YTHDF3 mRNA did not result in significant changes in FGF2 mRNA levels. We thus hypothesized that FGF2 potentially exhibits its effects through the recognition of the YTH domain on YTHDF3. Further exploration of this interaction was undertaken through Co-IP experiments to examine the protein-protein interactions between FGF2 and YTHDF3. As anticipated, the specific protein interaction between FGF2 and the mutated YTH domain of YTHDF3 was weakened. Additionally, utilizing the SRAMP tool (http://www.cuilab.cn/sramp/) (Zhou et al., 2016), we predicted potential m^6^A binding sites in the coding sequence (CDS) region of FGF2. RNA pull-down experiments confirmed that the binding between YTHDF3 and FGF2 mRNA was abolished when these predicted sites were mutated individually or in combination. These findings provide further support for our hypothesis. Furthermore, considering previous research indicating the role of YTHDF3 in protein stability regulation (Li et al., 2017), we investigated whether YTHDF3 modulates the stability of FGF2 protein. Through cycloheximide (CHX) and MG132 treatments, we demonstrated that YTHDF3 indeed influences the stability of FGF2 protein. Low levels of FGF2 inhibit the malignant progression of breast cancer cells. Nonetheless, it is important to acknowledge that YTHDF3 may regulate the expression of multiple genes in breast cancer, and its effects on breast cancer may be attributed to its broad impact on various targets. Further validation is necessary.

## 5 Conclusion

In summary, our research elucidates the critical role of YTHDF3 in breast cancer progression and unveils an intriguing m^6^A-dependent regulatory mechanism. The integrated network of YTHDF3 and its target gene FGF2 underscores a novel m^6^A-dependent gene regulatory mechanism within the realm of epigenetics. Moreover, the YTHDF3-m^6^A-FGF2 model suggests the potential for a new therapeutic strategy in breast cancer treatment through the suppression of YTHDF3 expression.

## Data Availability

The original contributions presented in the study are included in the article/Supplementary Material, further inquiries can be directed to the corresponding author.

## Abbreviations

BC, Breast Cancer; CDS, Coding sequence; CHX: Cycloheximide; Co-IP, Co-Immunoprecipitation; MeRIP-seq, Methylated RNA immunoprecipitation sequencing; m^6^A, N6-methyladenosine; MG132, Carbobenzoxy-Leu-Leu-leucinal; FGF2, Fibroblast growth factor 2; HR, Hazard ratio; IHC, Immunohistochemical; KO, Knockout; RIP, RNA immunoprecipitation; ROC, Receiver operating characteristic; RT-qPCR, quantitative real-time PCR; TCGA, The Cancer Genome Atlas; YTHDF3, YTH N6-methyladenosine RNA binding protein 3.
