# Supplementary material for: YTHDF3 modulates the progression of breast cancer cells by regulating FGF2 through m6A methylation
Source: Front Cell Dev Biol. 2024 Sep 20;12:1438515. doi: 10.3389/fcell.2024.1438515 (PMC11449838; doi:10.3389/fcell.2024.1438515)
Supplement: Supplementary file 2 [file Table1.DOCX]

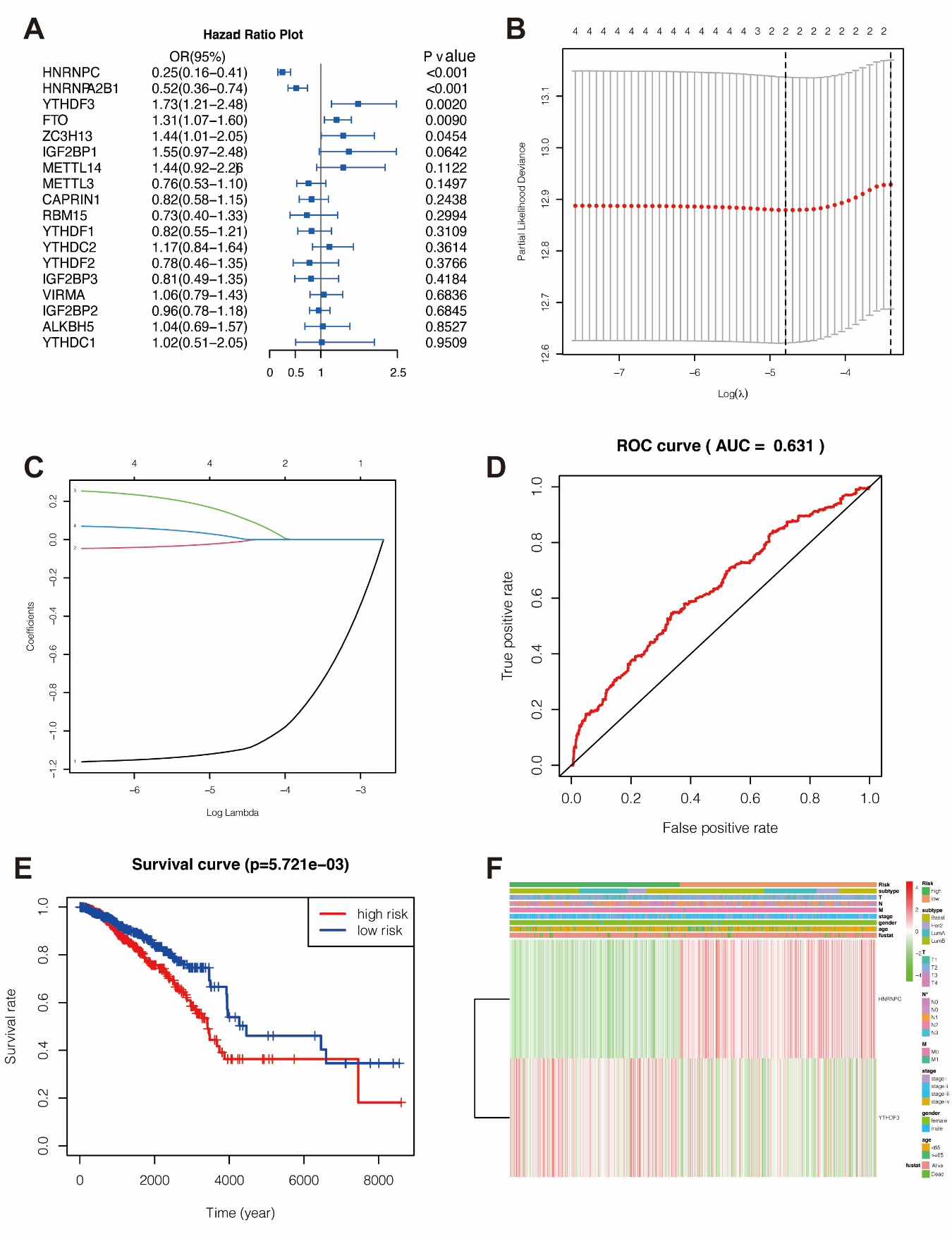


Figure S1. Selection of risk factors associated with m^6^A genes. (A) Calculation of 95% confidence intervals using univariate Cox regression; LASSO regression employed to determine the minimum standard (B) and coefficients (C). (D) Assessment of the predictive efficiency of the survival model based on risk feature grouping using Receiver Operating Characteristic (ROC) curve. (E) Analysis of survival curves for patients in the high- and low-risk groups. (F) Comparative analysis of clinical pathological features and the expression levels of YTHDF3 and HNRNPC based on risk feature grouping.
