# Supplementary material for: YTHDF3 modulates the progression of breast cancer cells by regulating FGF2 through m6A methylation
Source: Front Cell Dev Biol. 2024 Sep 20;12:1438515. doi: 10.3389/fcell.2024.1438515 (PMC11449838; doi:10.3389/fcell.2024.1438515)
Supplement: Supplementary file 3 [file Table2.DOCX]

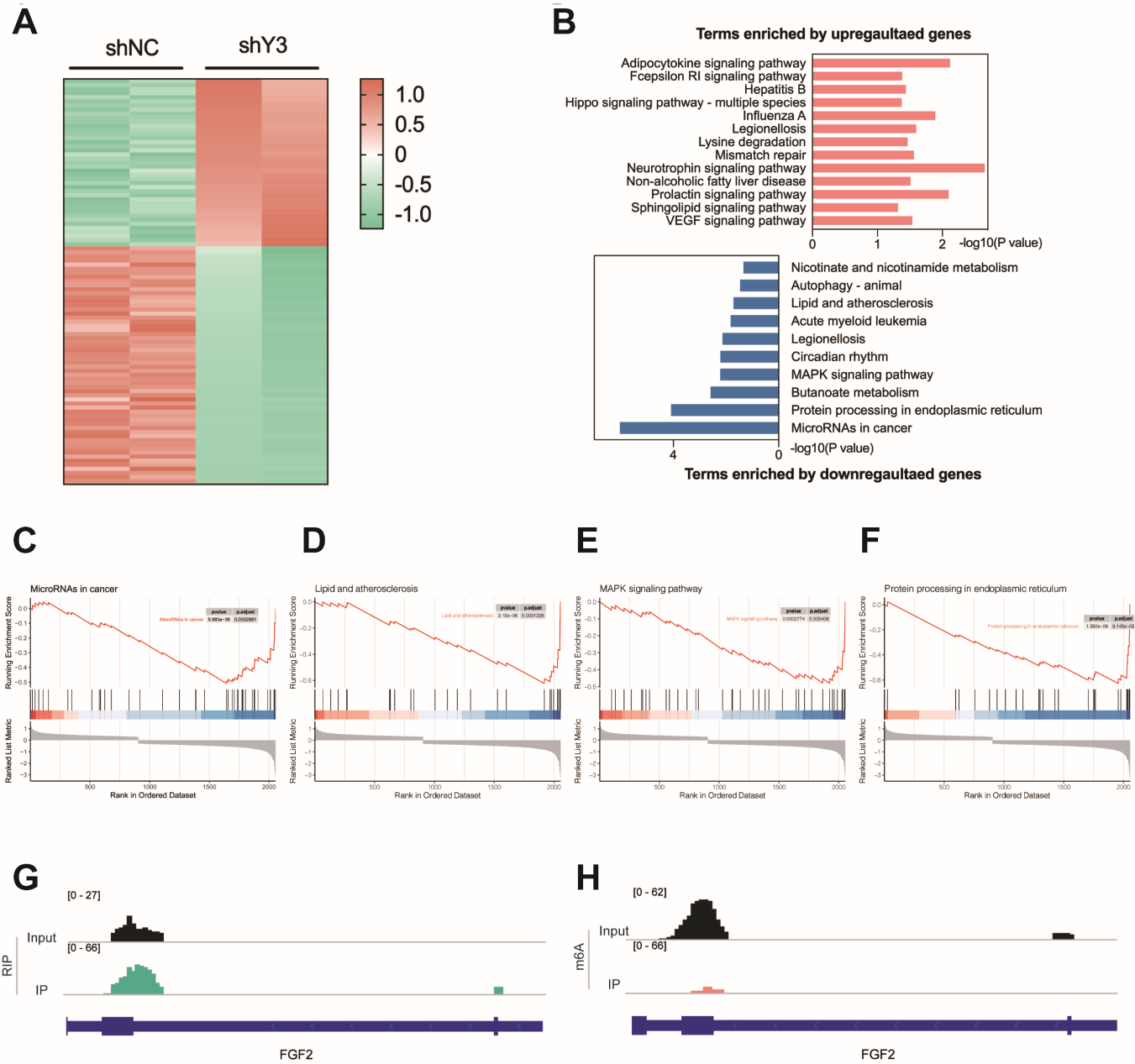


Figure S2. Identification of YTHDF3 Targets in Breast Cancer Cells. (A) Heatmap representing the differentially expressed genes (DEGs) identified through RNA-seq analysis. (B) GO enrichment analysis performed on the DEGs. (C-F) GSEA plots demonstrating the participation of YTHDF3-modulated DEGs in breast cancer cell pathways. (G-H) Distribution of m^6^A peaks and YTHDF3-binding peaks across FGF2 transcripts.
